# Second trimester amniotic fluid cytokine concentrations, *Ureaplasma* sp. colonisation status and sexual activity as predictors of preterm birth in Chinese and Australian women

**DOI:** 10.1186/1471-2393-14-340

**Published:** 2014-09-30

**Authors:** Matthew S Payne, Zhenhua Feng, Shaofu Li, Dorota A Doherty, Biyun Xu, Jie Li, Lenan Liu, Jeffrey A Keelan, Yi Hua Zhou, Jan E Dickinson, Yali Hu, John P Newnham

**Affiliations:** School of Women’s and Infants’ Health, The University of Western Australia, King Edward Memorial Hospital, 374 Bagot Road, Subiaco, 6008 Western Australia Australia; Department of Obstetrics and Gynaecology, Nanjing Drum Tower Hospital, The Affiliated Hospital of Nanjing University Medical School, Nanjing, China

**Keywords:** *Ureaplasma* sp, Preterm birth, Cytokines, Amniotic fluid, Sex in pregnancy

## Abstract

**Background:**

This study tested if second trimester amniotic fluid cytokine levels, *Ureaplasma* sp. colonisation and sexual activity predict preterm birth and explain the differential preterm birth rates in Chinese compared to Australian women.

**Methods:**

Amniotic fluid was collected by amniocentesis (Chinese 480, Australian 492). Cytokines were measured by multiplex assay and *Ureaplasma* sp. DNA was detected by PCR analysis. Lifestyle factors, including history of smoking and sexual activity during pregnancy, were obtained through completion of questionnaires upon recruitment to the study.

**Results:**

Inflammatory cytokine concentrations were poorly predictive of preterm birth. *Ureaplasma* sp. was detected in two of the Chinese pregnancies and none from Australia. Sexual activity was less frequent in Chinese, and was not associated with preterm birth or amniotic fluid findings in either population.

**Discussion:**

Second trimester amniocentesis for measurement of inflammatory markers and *Ureaplasma* sp. DNA was not indicative of risk of preterm birth, at least in these populations. The lower rate of preterm birth in China was not explained by differences in amniotic fluid inflammatory markers, *Ureaplasma* sp. colonisation, or sexual activity.

**Electronic supplementary material:**

The online version of this article (doi:10.1186/1471-2393-14-340) contains supplementary material, which is available to authorized users.

## Background

Preterm birth (PTB) is the leading cause of death and disability in young children in the developed world [1, 2]. In Australia, the rate of PTB is similar to the majority of developed countries at 7-9% and has been increasing over recent decades [3]. In contrast, the rate of PTB in China is believed to be less than 4%. Previously, we have reported that the PTB rate in Jiangsu Province, of which Nanjing is the capital, is 2.6-2.9% [4]. Further, we observed that migration of Chinese women from mainland China to westernised populations was associated with a subsequent increase in risk of PTB. Chinese women in Hong Kong without residency status had rates of 5-6%, increasing to 7.6% for those with residency status, while Chinese women in Western Australia had rates of 4.4% compared to 7-8% in Australian Caucasians [4].

The reasons underpinning the different rates of PTB in China and Australia are not understood. We previously speculated that lifestyle differences, particularly attitudes towards smoking and sexual activity during pregnancy, may be involved. A subsequent study by Zhang et al. [5] reported a significant association between sexual activity and PTB in Chinese women. However, evidence to support a similar association in western populations is conflicting [6]. Due to the multifactorial nature of PTB, there are numerous other factors likely to be involved, in particular the effects of infection and inflammation.

There is strong evidence that infection and inflammation are the leading causes of very early PTB and are also involved to a lesser extent in later PTB [7]. In terms of infection, numerous organisms have been associated with PTB. These range from common sexually transmitted disease-associated organisms such as *Chlamydia trachomatis* [8] to the large number of anaerobic genera associated with bacterial vaginosis [9]. However, intrauterine tissues from preterm pregnancies are most frequently colonised with *Ureaplasma* sp. and there is strong evidence from both animal models [10, 11] and human pregnancies [12, 13] for a causal relationship between this organism and PTB. In particular, several previous studies have suggested significant associations between the presence of *Ureaplasma* sp. in amniotic fluid and subsequent PTB [14–16]. For example, Yoon et al. [15] isolated *Ureaplasma* sp. from the amniotic fluid of 11/181 (6.1%) women in preterm labor and reported significantly higher rates of PTB within 48 h, 72 h and 7 days of amniocentesis for these women. *Ureaplasma* sp. colonisation of the vagina, the likely source of amniotic fluid infection, has also been widely examined for potential association with PTB; however, evidence for such an association has been inconsistent [17].

Measurements of second trimester amniotic fluid cytokine and chemokine levels have previously been explored to identify women at an elevated risk of PTB [18–20] and diagnose intrauterine infection [21, 22]. In particular, elevated levels of amniotic fluid interleukin-6 (IL-6) have been reported as predictive of intrauterine infection [21–24], as well as predictive of PTB in the absence of symptomatic clinical infection [18, 24]. In addition to IL-6, associations between infection/inflammation/PTB and a wide range of other amniotic fluid cytokines and chemokines have been described. Examples include interleukin-8 (IL-8) [20–22, 24], interleukin-1β (IL-1β) [21, 23], interleukin-10 (IL-10) [20, 21], monocyte chemotactic protein-1 (MCP-1) [25–27], matrix-metalloproteinase-8 (MMP-8) [18] and tumour necrosis factor-alpha (TNF-α) [21, 23].

It has been suggested that because intra-amniotic inflammation is heterogeneous, not all cases of elevated cytokine/chemokine levels can be predictive of adverse pregnancy outcome [19]. Further, racial factors may be involved. Peltier et al. [21] described differing pro and anti-inflammatory properties of amniotic fluid between European-Americans and African-Americans, as well as differing responses depending on the particular agonist involved. These authors suggested that assessment of biomarkers for prediction of pregnancy outcome may need to be customised according to race, in addition to the agonist responsible for causing the inflammation.

To our knowledge, there are no published reports describing the prevalence of intraamniotic *Ureaplasma* sp. colonisation in Chinese women, or comparing amniotic fluid cytokine/chemokine levels between Chinese and Western populations. We hypothesised that lower rates of *Ureaplasma* sp. colonisation of amniotic fluid, differences in the intraamniotic inflammatory response to such colonisation, and related lifestyle factors, may explain the apparent differences in preterm birth rates between the two populations.

The aims of this study were, therefore, to describe the levels of inflammatory cytokines IL-1β, IL-6, IL-10, TNF-α and the chemokine MCP-1 and colonisation rates of *Ureaplasma parvum* and *Ureaplasma urealyticum* in 2^nd^ trimester amniotic fluid samples from Chinese and Australian women; and to evaluate the association between these variables, lifestyle factors including smoking and sexual activity during pregnancy, and PTB. If individual, or a combination, of inflammatory and infectious markers were to be strong predictors of early PTB, amniocentesis early in the 2^nd^ trimester in cases suspected to be at high risk could become a useful clinical tool.

## Methods

### Subjects

The study consisted of two cohorts of women from whom amniotic fluid samples and clinical outcome data were available: 512 from Drum Tower Hospital, Nanjing, China and 564 from King Edward Memorial Hospital (KEMH), Perth, Western Australia.

Women with a singleton pregnancy were eligible for inclusion if they had been referred for genetic amniocentesis. In the Australian cohort, indications to conduct this procedure were primarily to exclude aneuploidy based on high risk findings at either first trimester screening (ultrasound measurement of fetal nuchal translucency and maternal biochemistry) or second trimester maternal serum screening (maternal biochemistry) with no apparent fetal structural anomalies. In the Chinese cohort, the indications for amniocentesis included advanced maternal age, nuchal translucency screening and maternal request. In all pregnancies, gestational age was determined by ultrasound imaging and biometry in the first or early second trimester. Participants were required to possess language fluency sufficient to understand the implications of participation. Cases subsequently shown to be complicated by aneuploidy were withdrawn and the amniotic fluid not analysed further.

All amniocenteses were performed between 15 and 20 weeks gestation. Written informed consent was obtained by the attending medical practitioner or midwife. Approval for the study was obtained from the ethics committees of Drum Tower Hospital, Nanjing, China (2011037), the Women and Newborn Health Service, Western Australia (1880/EW), and The University of Western Australia (RA/4/1/4784).

The preterm birth rate in the entire Chinese sample of 1865 pregnancies was 3.4%, consistent with previous observations in this population [4]. In view of the cost of analyses, the Chinese group was enriched with cases of preterm birth; the final sample included all cases of preterm birth (63 cases) and a random selection of term births producing a sample of 512 cases. Of these, 32 cases were excluded due to inadequate follow-up data, leaving 480 for analysis.

564 consecutive cases in the Australian group were included in the study, of which 72 were lost to follow-up leaving 492 for analysis.

### Questionnaire

Prior to amniocentesis, women were invited to complete a de-identified lifestyle questionnaire in a private setting. The questionnaire inquired about smoking practices as yes or no, and then sought to quantify the number of cigarettes smoked each day in those who declared themselves to be smokers. Sexual activity during pregnancy was assessed by inquiring about the number of episodes of sexual intercourse during the pregnancy. Responses to this question were categorised as: 1) On most days/daily; 2) 2–3 times each week; 3) About once each week; 4) Less than once each week; and 5) Not at all. Information regarding any vaginal bleeding in the two weeks preceding amniocentesis was also recorded.

### Pregnancy outcome data

Pregnancy outcome data were accessed by review of the medical records after completion of the pregnancy.

### Sample collection

Amniocenteses were conducted by medical specialists under ultrasound guidance at 15–20 weeks gestational age (GA). Three millilitres of amniotic fluid were aspirated in addition to the 10–15 mL collected for clinical measurement and subsequently dispensed into 1 mL aliquots in sterile cryovials prior to storage at −80°C.

### Sample preparation and processing - Chinese pregnancies

From each participant, 1.5 mL of amniotic fluid was centrifuged at 15,000 × *g* for 10 min. Supernatant was removed and frozen at −20°C or −80°C for cytokine analyses. The pellet and approximately 100 μL of residual supernatant was subsequently frozen at −20°C for later DNA extraction.

### DNA extraction

Pelleted amniotic fluid samples were thawed and treated with proteinase K (0.2 mg/mL) (Sigma, Saint Louis, USA) at 50°C for 3 h. DNA was subsequently extracted using two phenol-chloroform separations and rinsed once with 70% ethanol following centrifugation at 10,000 *× g*. DNA was eluted in 25 μL Tris-EDTA buffer and frozen at −20°C prior to PCR analysis.

### PCR analysis

For detection of *Ureaplasma* sp. DNA, amniotic fluid DNA was screened using PCR analysis. Analysis was performed using two end-point PCR assays targeting the multiple-banded antigen and urease genes of *U. parvum* and *U. urealyticum* as described by Stellrecht et al. [28]. For each assay, reaction mixtures consisted of: 1.25 units of Taq DNA polymerase (Takara, Dalian, China), 12.5 μL template DNA, 2 mM MgCl_2,_ 0.2 mM dNTPs and 0.3 μM primers; water was added to a final volume of 50 μL. PCR cycling conditions consisted of an initial denaturation at 94°C for 3 min followed by 35 cycles of 94°C for 30 s, 55°C for 30 s and 72°C for 40 s. A final extension step was performed at 72°C for 5 min. PCR products were visualised by electrophoresis on a 1.5% agarose gel stained with ethidium bromide, and were photographed using an ultra-violet image analysis system (UVP, Upland, CA).

### Sequencing of MBA amplicons

For positive samples, to confirm species identity, MBA amplicons were purified using TIANquick Midi Purification kits (Tiangen Biotech, Beijing, China) and directly sequenced on an ABI Prism 3130 sequencer (Applied Biosystems, Hitachi, Tokyo, Japan) using BigDye Terminator v3.1 chemistry (Applied Biosystems, Foster, CA) as per manufacturer’s instructions. Sequences were analysed using the Basic Local Alignment Search Tool (BLAST) (http://blast.ncbi.nlm.nih.gov/Blast.cgi) for comparison against already established *Ureaplasma* sp. MBA gene sequences.

### Sample preparation and processing - Australian pregnancies

For each participant, 1 mL of amniotic fluid was thawed, centrifuged at 16,100 × *g,* 4°C for 30 min and the supernatant transferred to a 1.5 mL microfuge tube, placed on ice and assayed immediately for cytokine analyses. The pellet and approximately 100 μL of residual supernatant were then frozen at −80°C until DNA extraction.

### DNA extraction

Amniotic fluid pellets were thawed and the total volume adjusted to approximately 250 μL with PBS (Sigma Aldrich, St Louis MO). DNA was extracted using the Versant Sample Preparation Kit 1.0 (Siemens AG, Erlangen, Germany) on the Kingfisher Duo automated extraction platform (Thermo Fisher Scientific, Vic, Australia) as per manufacturer’s recommendations, and frozen at −20°C prior to analysis by real-time PCR. A positive extraction control consisting of approximately 250 colour changing units (CCU) each of *U. parvum* and *U. urealyticum* was included in all runs.

### Real-time PCR analysis

For detection of *Ureaplasma* sp. DNA, amniotic fluid DNA was analysed using a real-time PCR assay targeting the urease gene of *U. parvum* and *U. urealyticum* as described by Yi et al. [29], adapted for use on a ViiA7 real-time PCR thermocycler (Life Technologies, Carlsbad, California, USA). Reaction mixtures (final volume or concentration) consisted of: 1X Taqman FAST Advanced Master Mix (Life Technologies), 0.9 μM primers UU1613F and UU1524R (Life Technologies), 0.25 μM probes UU-parvo (FAM) and UU-T960 (VIC) (Life Technologies), 5 μL of template DNA and nuclease-free water (Ambion) to a final volume of 20 μL. PCR cycling conditions consisted of an initial denaturation/Taq activation at 95°C for 20 s, followed by 40 quantification cycles of 95°C for 1 s and 60°C for 20 s (data acquiring).

### Cytokine/chemokine analysis – Chinese and Australian pregnancies

Concentrations of IL-1β, IL-6, IL-10, TNF-α and MCP-1 were determined by multiplex assay using the Milliplex MAGPIX system (EMD-Millipore/Merck KGaA, Darmstadt, Germany) as per manufacturer’s instructions. Briefly, individual wells of 96-well plates were loaded in duplicate with either: 25 μL of prepared standard solution, 25 μL of amniotic fluid supernatant or 25 μL of quality control. 25 μL premixed antibody-coated beads were then added to all wells. Plates were incubated at 500 RPM, 4°C, overnight in the dark and then washed twice with 200 μL wash buffer followed by magnetic precipitation prior to the addition of 25 μL of detection antibody. Plates were then incubated at room temperature for one hour, 300 RPM in the dark. 25 μL of streptavidin-phycoerythrin conjugate was subsequently added to each well and the plate again incubated at room temperature for 30 min, 300 RPM in the dark. Plates were washed twice more with wash buffer and following magnetic precipitation the labelled beads were resuspended in 150 μL of drive fluid for measurement in the MAGPIX instrument.

The calibration curve range for all cytokines/chemokines was initially 3.2 – 10,000 pg/mL according to the manufacturer’s instructions. Samples which were below this limit were subsequently re-assayed with an extended standard curve range that provided a minimum level of detection of 0.64 pg/mL. To assess comparability between assays performed in China and Australia, two sets of commercial QCs (Randox Life Sciences, County Antrim, Northern Ireland) representing low and high values, respectively, were measured with each run. The same lot numbers of QCs and Milliplex MAGPIX assays were employed by both laboratories to minimise variability. In order to ensure comparability of the cytokine values between the Chinese and Australian groups, the values were adjusted to normalise the quality control values (Additional file 1: Table S1) across both groups.

### Statistical analyses

Adjustment factors were required in this study to account for the fact that all laboratory measurements had be done in the country of origin of the sample as it is illegal to import or remove human samples to or from China.

Continuous data were summarised using means and standard deviations or medians, interquartile ranges (IQR) and ranges (R), as appropriate for data normality. Categorical outcomes were summarised using frequency distributions. Univariable comparisons of cytokines/chemokines and lifestyle and pregnancy features/outcomes were based on Mann–Whitney or Kruskal-Wallis tests and Chi-square or Fisher exact tests for continuous and categorical data as required. Apart from the comparisons of cytokine/chemokine levels between Chinese and Australian pregnancies, all analyses were performed using Chinese pregnancy data or Australian pregnancy data alone. The effects of cytokine/chemokine levels at amniocentesis on gestational age at delivery and PTB were investigated using Cox proportional hazards regression with hazard ratios (HR) and logistic regression analysis with odds ratios (OR) and their 95% confidence intervals (CI) used as summaries. Multivariable effects of cytokines/chemokines were examined while considering adjustments for other relevant risk factors without model overfitting. SPSS version 20 (SPSS IBM, New York, USA) statistical software was used for data analysis. P-values < 0.05 were considered statistically significant.

## Results

### Subjects

Data from the 480 Chinese and 492 Australian women and their pregnancies are shown in Table 1. The preterm birth rate in the Chinese group, after enrichment, was 13.1% (increased from an initial rate of 3.4%) and in the Australian group was 10.6%.

**Table 1:** **Maternal characteristics of the Chinese and Australian pregnancies**

	China (n = 480)	Australia (n = 492)
Nulliparity	308 (64.2%)	170 (34.6%)
Maternal age; median (interquartile range; range)	29 (26–36; 17–49)	35 (31–39; 17–48)
Ethnicity		
Chinese	480	N/A
Caucasian	N/A	282
Asian	N/A	9
Aboriginal	N/A	85
Did not answer	N/A	116
Smoking in pregnancy	3 (1%)	66 (13.4%)
Preterm birth	63 (13.1%)^1^	52 (10.6%)
Threatened abortion	1 (0.2%)	15 (3.0%)
Gestational age (GA), preterm (min – max)	28-36	21-36
<28 weeks	-	6 (1.2%)
28-32 weeks	5 (1.0%)	3 (0.6%)
33-36 weeks	58 (12.1%)	43 (8.7%)
37+ weeks	417 (86.9%)	440 (89.4%)
Stillbirth	1	8
Premature rupture of membranes (PROM)	77 (16.0%)	68 (13.8%)
Preterm PROM	27 (5.6%)	19 (3.9%)

^1^The preterm birth rate in the Chinese sample was enriched from an initial rate of 3.4%.

### Detection/quantitation of cytokines

Minimum, median (IQR) and maximum cytokine values detected in amniotic fluid from the Chinese and Australian pregnancies are presented in Table 2. Levels of IL-10 (median 7.3 *vs*. 6.3 pg/mL), MCP-1 (median 1145 *vs*. 811.1 pg/mL) and TNF-α (median 6.5 *vs*. 5.6 pg/mL) were significantly lower in Australian pregnancies (*P* < 0.001 for each variable). There were no significant differences in IL-1β and IL-6 between Chinese and Australian pregnancies.

**Table 2:** **Amniotic fluid cytokine concentrations (median and interquartile range [range]) in Chinese and Australian pregnancies**

	Cytokine (pg/mL)				
	IL-1β	IL-6	IL-10	MCP-1	TNF-α
**China**	0.8 (0.6 - 1.4)	74.4 (46.8 - 154.1)	7.3 (4.9 - 11)	1145 (889 - 1462)	6.5 (5.2 - 7.9)
	[range: 0.6 - 6.6]	[range: 9.5 - 1675]	[range: 2.9 - 417]	[range: 312 - 7635]	[range: 2.4 - 81.0]
	N = 479	N = 480	N = 480	N = 480	N = 480
**Australia**	0.9 (0.6 - 1.4)	91.0 (39.7 - 206)	6.3 (3.5 - 9.0)	811 (559 - 1114)	5.6 (3.6 - 7.4)
	[range: 0.6 - 6.5]	[range: 32 - 6402]	[range: 3.2 - 93.1]	[range: 3.0 - 9300]	[range: 3.2 - 19.1]
	N = 436	N = 421	N = 449	N = 438	N = 451

No significant differences were found between cytokine levels and either maternal age or fetal sex within pregnancies from either country, although within the Chinese pregnancies median IL-10 values tended to be higher in males (9.1 *vs*. 7.9 pg/mL).

### Gestational age at delivery/preterm birth

**Figure 1:**
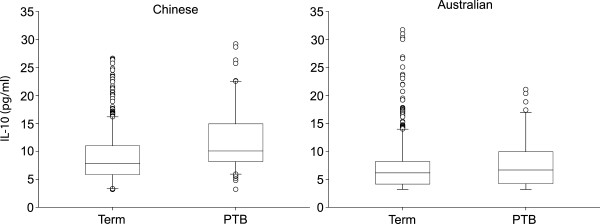
**Box and whisker plots illustrating the associations between amniotic fluid IL-10 levels and PTB in Chinese and Australian pregnancies.** PTB was significantly associated with increased amniotic fluid IL-10 levels in Chinese but not Australian pregnancies (*P* = 0.009).

Within Chinese pregnancies, elevated IL-10 levels (defined as >10.1 pg/mL) were significantly associated with an earlier gestational age at delivery (*P* = 0.012; HR = 1.27; 95% CI: 1.05-1.53). The median levels of IL-10 were also significantly higher in Chinese but not Australian pregnancies for PTB defined as birth before 37 completed weeks (*P* = 0.009; OR = 2.25; 95% CI: 1.23-4.13) (Figure 1). In amniotic fluid from either country, there was no association between levels of any other cytokine and either delivery at an early gestational age or PTB.

There was a significant association between elevated IL-10 levels (i.e. >10.1 pg/mL) in Chinese pregnancies and pre-labour rupture of membranes (PROM) (*P* = 0.008; HR = 1.84; 95% CI: 1.17-2.88). High levels of IL-10 within Australian pregnancies were not associated with PROM (*P* = 0.578), while elevated TNF-α levels were paradoxically associated with decreased likelihood of PROM (P = 0.031; HR = 0.46; 95% CI: 0.22-0.93). No other cytokines were associated with PROM in pregnancies from either country.

### Smoking during pregnancy

Smoking during pregnancy was rare amongst Chinese pregnancies (1%) with only three women reported to be smokers. Amongst Australian pregnancies 13.4% of women smoked. The rate of PTB in Australian smokers was 16.7% compared with 9.3% in non-smokers. In the Australian pregnancies, smoking was significantly associated with increased amniotic fluid IL-6 levels (median 171.5 *vs*. 89.0 pg/mL)(*P* = 0.001).

### Sexual intercourse during pregnancy

**Figure 2:**
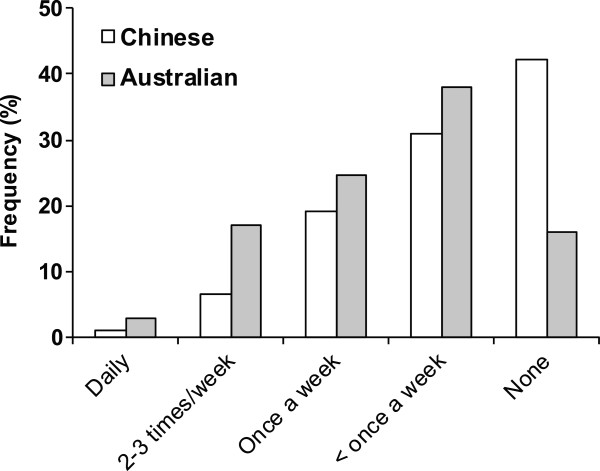
**Reported frequency of sexual intercourse during pregnancy in Chinese and Australian women.** The rate reported by Chinese women was significantly less than in Australian women (*P* < 0.001).

**Figure 3:**
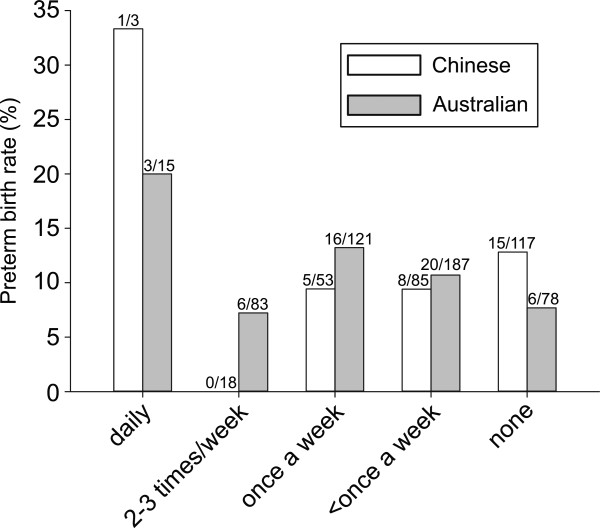
**Reported frequency of sexual intercourse during pregnancy and rates of PTB in Chinese and Australian women.** There was no significant association between these rates and PTB in either group.

**Figure 4:**
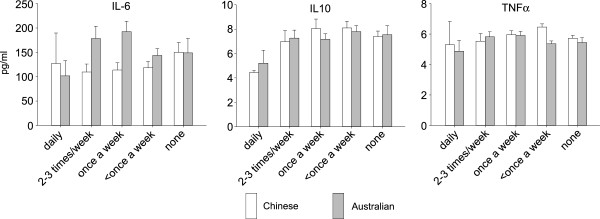
**Reported frequency of sexual intercourse during pregnancy and amniotic fluid cytokine levels in Chinese and Australian pregnancies.** There were no associations between sexual activity and cytokine levels in either group.

Rates of sexual intercourse reported by Chinese women were less frequent than rates reported by Australian women (Figure 2) (*P* <0.001). There was no significant association between rates of sexual intercourse and PTB in either Chinese or Australian women (*P* = 0.329 and *P* = 0.407 respectively) (Figure 3). Further, after combining the data from both groups, there remained no statistical association between frequency of sexual intercourse and PTB. There was also no significant association between frequency of sexual intercourse during pregnancy and amniotic fluid cytokine levels in pregnancies from either country (all *P* >0.05) (Figure 4). Data were not available from 203 Chinese women of whom 195 were administered the questionnaire and eight chose not to respond. Eight Australian women also declined to answer this question.

### Threatened abortion

Threatened abortion in the two weeks before the amniocentesis was reported in only one case of the Chinese pregnancies. Within the 15 Australian pregnancies complicated by recent threatened abortion, there was a significant association with elevated levels of IL-6 (median 285.7 vs. 88.6 pg/mL; *P* = 0.024) and IL-10 (median 11.6 vs. 6.1 pg/mL; *P* = 0.001). Levels of IL-1β were also increased in these cases (median 1.1 vs. 0.9 pg/mL; *P* = 0.05).

### Detection of *Ureaplasma*sp. DNA and preterm birth/cytokine association

Two amniotic fluid samples were positive for *U. parvum* DNA from Chinese pregnancies. Both women from whom these samples were obtained delivered preterm, one at 29 weeks and the other at 34 weeks gestation. Both these women had significantly elevated cytokine levels for every cytokine measured relative to mean levels (Table 3). *U. urealyticum* DNA was not detected in any of the Chinese samples. In the amniotic fluid samples from Australian women, there were no cases in which *U. parvum* or *U. urealyticum* was detected. Extraction controls were positive in all assays.

**Table 3:** **Comparison of median cytokine levels in amniotic fluid from two** ***Ureaplasma*** **sp. DNA positive Chinese women who delivered preterm**

	Cytokine (pg/mL)				
	IL-1β	IL-6	IL-10	MCP-1	TNF-α
**Participant 1**	2.59	1675.5	417.1	5161	81.0
**Participant 2**	1.89	435.9	238.1	7635	61.1
**All group median**	0.8	74.4	7.3	1145	6.5

## Discussion

In this study, we have explored the hypothesis that amniotic fluid cytokines/chemokines and *Ureaplasma* sp. colonisation sampled by amniocentesis early in the second trimester may be useful to predict PTB, thus enabling therapeutic interventions aimed at prevention. In addition, we have compared amniotic fluid from populations of pregnant women in China and Australia to investigate whether either of these factors can help to explain the major differences seen in rates of PTB in these countries, in particular why westernisation appears to be associated with an increased rate of PTB in Chinese women. Amniotic fluid inflammatory cytokines/chemokines were in general not predictive of PTB, although elevated IL-10 levels were associated with PTB and PROM in Chinese pregnancies. In Australian pregnancies, low levels of TNF-α were unexpectedly associated with subsequent PROM. The differential responses between these two populations most likely represent a combination of racially-specific factors and response to intrauterine agonists. Racial differences in amniotic fluid cytokine profiles have been described previously by Velez et al. [30] who reported that within European-American and African-American women there appears to be differential genetic control of amniotic fluid levels of IL-6. Subsequently, Menon et al. [31] showed a significant association between IL-6 levels and PTB in Caucasians, but not in African-Americans. In addition, these authors found no association between amniotic fluid IL-10 levels and PTB, whereas in the present study we observed a small but significant association between amniotic fluid IL-10 levels and PTB in Chinese pregnancies. This observation suggests that within Chinese women, inflammation-driven PTB is likely triggered through production of different pro- and anti-inflammatory cytokines. Such a possibility has been suggested by data from another study by Menon et al. [32] with respect to African-Americans and Caucasian-Americans. These authors reported higher amniotic fluid levels of IL-1β and IL-8 in both groups, but elevated IL-1β levels were only associated with PTB in African-Americans. Most recently, these authors [33] examined the association between single nucleotide polymorphisms in cytokine and cytokine-related genes with amniotic fluid levels of IL-1β, IL-8 and IL-10 from term and preterm births amongst African-Americans and Caucasian-Americans. It was concluded that genetic regulation of cytokine levels during pregnancy was likely related to ethnicity and, in particular, that amniotic fluid cytokine levels were linked to interactions between PTB and genotype in African-Americans, but less in Caucasian-Americans. An examination of the nucleotide composition of cytokine-related genes during pregnancy within Chinese and Australian women would be of interest to potentially explain the difference in levels observed in the present study.

In addition to the direct impact of racially-specific genetic factors on cytokine regulation within the amniotic fluid, recent research has also reported racially-specific amniotic fluid cytokine responses to different intrauterine agonists. Using cultured fetal membranes from European-American and African-American women exposed to a range of bacteria, Peltier et al. [21] demonstrated organism-specific cytokine responses that differed between the two cohorts. For example, IL-1β and TNF-α production was up-regulated in the presence of lipopolysaccharide (LPS), *Escherichia coli* and *Gardnerella vaginalis* relative to control. In addition, within their study, African-Americans showed double the IL-1β response to *E. coli* compared with European–Americans. In contrast, IL-8 production in response to LPS was significantly up-regulated in European-Americans compared to African-Americans. The results of the present study support the view that race should be considered when interpreting cytokine responses in PTB prediction.

In the present study, we detected *Ureaplasma* sp. DNA in only two of the 480 amniotic fluid samples from Chinese women and did not detect any in the 492 amniotic fluid samples from Australian women. The two Chinese cases where *Ureaplasma* sp. DNA was detected were associated with significantly elevated levels of all cytokines measured. A similar result was reported by Jacobssen et al. [34], who described an association between TNF-α levels and presence of *Ureaplasma* sp. DNA in amniotic fluid from women with PTL and PPROM. However, in contrast, this study found no association between levels of IL-1β, IL-6 and IL-10 and detection of *Ureaplasma* sp. DNA.

Differences in the rates of *Ureaplasma* sp. DNA detection in the current study are unlikely to have arisen from the differences in the DNA extraction and PCR methodology utilised by the two collaborative laboratories. Due to the lack of a cell wall, lysis of *Ureaplasma* sp. cells is readily achieved and both PCR assays were validated for *Ureaplasma* sp. detection. It is possible that due to the use of end-point PCR for screening of Chinese amniotic fluid samples, trace levels of *Ureaplasma* sp. DNA may not have been detected; such amounts, however, are unlikely to be clinically relevant. The very low detection rate of *Ureaplasma* sp. DNA in both sets of mid-trimester amniotic fluid samples is in stark contrast to previously reported observations in other populations. For example, several previous studies have reported *Ureaplasma* sp. within the amniotic fluid of women with preterm labour (PTL) and/or preterm premature rupture of membranes (PPROM), with detection rates ranging from 3.3% [35] to 63% [36]. Of particular significance is the case–control study by Gerber et al. [36], who described a significant association between presence of *Ureaplasma* sp. DNA in second trimester amniotic fluid and subsequent PTL. Similar associations have been described by Yoon et al. [14, 15] and Oh et al. [16] in case-specific studies. However, in contrast, within amniotic fluid from women who delivered at term, detection rates for *Ureaplasma* sp. ranging from 0% [37] to 5.3% [36] have been described.

As the current study is the first of its kind to examine amniotic fluid from both Chinese and Australian populations, there are no geographically comparable data against which our data can be compared. However, a previous study on an Australian population of pregnant women described the prevalence rate of *Ureaplasma* sp. within vaginal and endocervical samples as 57% and 17% respectively [34]. This is comparable to that reported in a range of vaginal prevalence studies conducted on different geographical cohorts of pregnant women [17]. The rate of colonisation in cervical swabs, however, is somewhat lower than that reported previously in a Chinese population. Kong et al. [38] collected 400 cervical swabs from sex workers (200), women attending a sexually-transmitted infection (STI) clinic (100) and pregnant women (100), and detected *Ureaplasma* sp. in 185/400 (46.3%) swabs. Interestingly, *Ureaplasma* sp. was detected significantly more frequently in swabs from pregnant women (58%) compared to sex workers or women attending the STI clinic (42.3%). These studies at the very least provide evidence that *Ureaplasma* sp. have the potential to invade the amniotic cavity in both Australian and Chinese women, based upon the theory of vaginal ascension [7]. Our results instead suggest that if *Ureaplasma* sp. do ascend into the amniotic cavity during pregnancy in these women, then they do so primarily at a time point after 20 weeks’ gestation. Taking this into account, particularly within the Australian setting, amniocentesis prior to 20 weeks gestation for detection of *Ureaplasma* sp. as an early predictive marker of PTB is unlikely to be a useful investigational tool. Unfortunately, it is rarely clinically possible to obtain amniotic fluid samples opportunistically after 20 weeks gestation as is the case with genetic amniocentesis in the early second trimester [39].

Our study observed significantly increased levels of amniotic fluid IL-6 in Australia women who smoked during pregnancy. Although it is well established that smoking during pregnancy is associated with PTB [40, 41], the effect of smoking on inflammatory responses within the amniotic cavity has been relatively unexplored. Previous studies have shown that smoking does influence cytokine levels in other bodily fluids. For example, Sazak et al. [42] demonstrated increased levels of erythropoietin within the umbilical cord blood of infants born to smokers and suggested that this finding may indicate a risk of fetal hypoxia and growth restriction. In contrast, Etem Piskin et al. [43] reported that levels of some cytokines in colostrum and breast milk were significantly lower in smokers.

Within Australian pregnancies we observed a significant association between elevated levels of IL-6 and IL-10 and vaginal bleeding within the previous two weeks. A recent study by Bamberg et al. [44] found that elevated amniotic fluid levels at 15–20 weeks gestation of IL-6, IL-8 and TNF-α were not predictive of spontaneous abortion in a study of 298 women with uncomplicated singleton pregnancies. To the best of our knowledge, our observation of elevated IL-10 levels following threatened abortion has not been described previously.

We previously suggested that differences in frequency of sexual intercourse between Chinese and Caucasian women in pregnancy may help to explain some of the disparity in PTB rates between the two groups [4]. While the current study demonstrated a significantly higher rate of sexual activity during pregnancy amongst Australian women, there was no evidence to suggest any association between frequency of sexual intercourse during pregnancy and either PTB or any individual amniotic fluid cytokine level. This result is similar to previous studies which either found no association between sexual activity during pregnancy and recurrent PTB [45] or actually described a reduced risk of PTB following intercourse during late pregnancy [6]. These data are all in contrast to the data presented by Zhang et al. [5] however, which suggested an almost two-fold increased risk of PTB in Chinese women who had sexual intercourse during pregnancy. It is important to note, however, that this study did not document the frequency of intercourse; it instead documented whether intercourse occurred at all during pregnancy. It also attempted to ascertain during what trimesters this occurred but did not receive a high compliance rate with this question (32.1% of subjects). To our knowledge, our study is the first of its kind to document sexual activity during pregnancy alongside levels of amniotic fluid inflammatory cytokines as potential indicators for risk of inflammation-driven PTB.

The present study is the first of which we are aware to compare data from Chinese and Western pregnancies. We have attempted to standardise all measurements to provide consistency between the two groups. Analyses, however, were complicated by two issues. First, it is illegal to remove or import any human samples from or into China, precluding the opportunity to conduct the measurements in one of the collaborating laboratories. Hence, we used identical measurement systems for amniotic fluid cytokines and comparable PCR methods for detection of *Ureaplasma* sp. DNA. Second, the low rate of PTB in the Chinese population required us to retrospectively enrich the sample to increase the rate of PTB.

## Conclusions

In conclusion, this study has shown that measurement of amniotic fluid inflammatory markers and detection of *Ureaplasma* sp. DNA in samples obtained early in the second trimester are not clinically useful as predictors of risk of preterm birth. The rarity of *Ureaplasma* sp. DNA in these two populations of pregnant women is in marked contrast to findings from other populations, highlighting the importance of conducting research in different countries and population groups. Sexual intercourse during pregnancy was less frequent amongst Chinese women and was not correlated with amniotic fluid inflammatory markers, presence of *Ureaplasma* sp. DNA or risk of PTB. These findings do not support the hypothesis that sexual activity in pregnancy is a cause of preterm birth.

## Authors’ information

Matthew S Payne and Zhenhua Feng are co-first authors; Yali Hu and John P Newnham are co-senior authors.

## Electronic supplementary material

Additional file 1: Table S1: Quality control (QC) values for all cytokines from the Chinese and Australian laboratories (mean ± standard deviation); data are from n=17 Chinese assays and n=4-7 Australian assays. The differences between the laboratories were not statistically significant. (DOCX 14 KB)
