# Supplementary material for: Second trimester amniotic fluid cytokine concentrations, Ureaplasma sp. colonisation status and sexual activity as predictors of preterm birth in Chinese and Australian women
Source: BMC Pregnancy Childbirth. 2014 Sep 30;14:340. doi: 10.1186/1471-2393-14-340 (PMC4261245; doi:10.1186/1471-2393-14-340)
Supplement: Supplementary file 1 — Additional file 1: Table S1: Quality control (QC) values for all cytokines from the Chinese and Australian laboratories (mean ± standard deviation); data are from n=17 Chinese assays and n=4-7 Australian assays. The differences between the laboratories were not statistically significant. (DOCX 14 KB) [file 12884_2014_1212_MOESM1_ESM.docx]

Additional file 1: Table S1 **- Quality control (QC) values for all cytokines from the Chinese and Australian laboratories (mean ± standard deviation);** data are from n=17 Chinese assays and n=4-7 Australian assays. The differences between the laboratories were not statistically significant.

|  | Mean ± sd cytokine concentration (pg/mL) | | | | |
| --- | --- | --- | --- | --- | --- |
|  | **IL-1β** | **IL-6** | **IL-10** | **MCP-1** | **TNF-α** |
| Low QC (China) | 13.88 ± 1.0 | 8.17 ± 0.78 | 51.7 ± 3.77 | 211.4 ± 13.8 | 15.9 ± 1.3 |
| Low QC (Australia) | 12.23 ± 1.5 | 5.14 ± 0.9 | 45.77 ± 3.2 | 188.1± 19.3 | 12.1 ± 1.7 |
| High QC (China) | 160.6 ± 10.4 | 246.7 ± 19.1 | 873.3 ± 72 | 1800 ± 95.8 | 286.6 ± 22.3 |
| High QC (Australia) | 157.1 ± 13.9 | 216.8 ± 19.4 | 907.2 ± 82.7 | 1925.5 ± 158.8 | 270.3 ± 10.9 |
